# Upregulation of AXL and β-catenin in chronic lymphocytic leukemia cells cultured with bone marrow stroma cells is associated with enhanced drug resistance

**DOI:** 10.1038/s41408-021-00426-2

**Published:** 2021-02-18

**Authors:** Sutapa Sinha, Charla R. Secreto, Justin C. Boysen, Connie Lesnick, Zhiquan Wang, Wei Ding, Timothy G. Call, Saad J. Kenderian, Sameer A. Parikh, Steven L. Warner, David J. Bearss, Asish K. Ghosh, Neil E. Kay

**Affiliations:** 1 Division of Hematology, Mayo Clinic, Rochester, MN USA; 2 Tolero Pharmaceuticals, Inc., Lehi, UT USA; 3 Stephenson Cancer Center and Department of Pathology, The University of Oklahoma Health Sciences Center, Oklahoma City, OK USA

**Keywords:** Cancer microenvironment, Cancer therapy

Despite the advent of even more effective therapies, Chronic Lymphocytic Leukemia (CLL) is still incurable and patients often develop drug resistance. We and others have found that bone marrow stromal cells (BMSCs) are excellent models for assessing the mechanism(s) by which stroma cells nurture CLL B-cells and we have shown that BMSC protects leukemic B-cells from spontaneous and drug-induced apoptosis^1^. The leukemic B-cell derives survival signals from stromal cells and the bone marrow site is able to harbor residual leukemic B-cells protected from chemotherapy. Prior evidence indicates that the facilitation of residual disease burden may be a key pathway to clonal evolution and ultimate clinical relapses difficult to treat in CLL^2^.

To further delineate additional resistance mechanisms, we evaluated alteration of critical survival pathways including AXL^3,4^, in leukemic B-cells upon co-culture with BMSCs. All experimental details are provided in the supplement. We co-cultured primary CLL B-cells (Supplementary Table 1) with BMSCs derived from healthy subjects or untreated CLL patients (Supplementary Table 2) and compared them to CLL B-cells cultured alone for 48 h. CLL B-cells were separated from BMSCs and analyzed by western blot (WB) analysis. A significant increase of AXL expression in post co-cultured CLL B-cells were detected compared to CLL B-cells cultured alone (Fig. 1A). Further analysis also found an increased accumulation of β-catenin in post co-cultured leukemic B-cells from basal levels (Fig. 1A). However, we could not detect any significant alteration of cell surface AXL levels on these leukemic B-cells by flow analysis (Fig. 1B, C), indicating an increase in AXL expression restricted to the cytoplasm. Additionally, we tested other malignant cell types and observed a significant increase of both AXL and β-catenin in B lymphoma cell lines (Mino, Raji, and SU-DHL4) upon their co-culture with BMSCs (Fig. 1D). Furthermore, by RT-PCR using specific sets of primers we observed a significant increase of AXL mRNA levels but not β-catenin mRNA levels in post co-cultured CLL B-cells compared to that in CLL B-cells cultured alone (Fig. 1E, F). Next, we examined the expression of AXL and β-catenin in CLL B-cells after their exposure to BMSCs for 48 h using either a direct co-culture method versus co-culturing CLL B-cells with BMSCs separated via transwells. Interestingly, we found increased expression of both AXL and β-catenin in CLL B-cells only when CLL B-cells were in direct contact with BMSCs but not when separated by transwells (Fig. 1G).

**Fig. 1:**
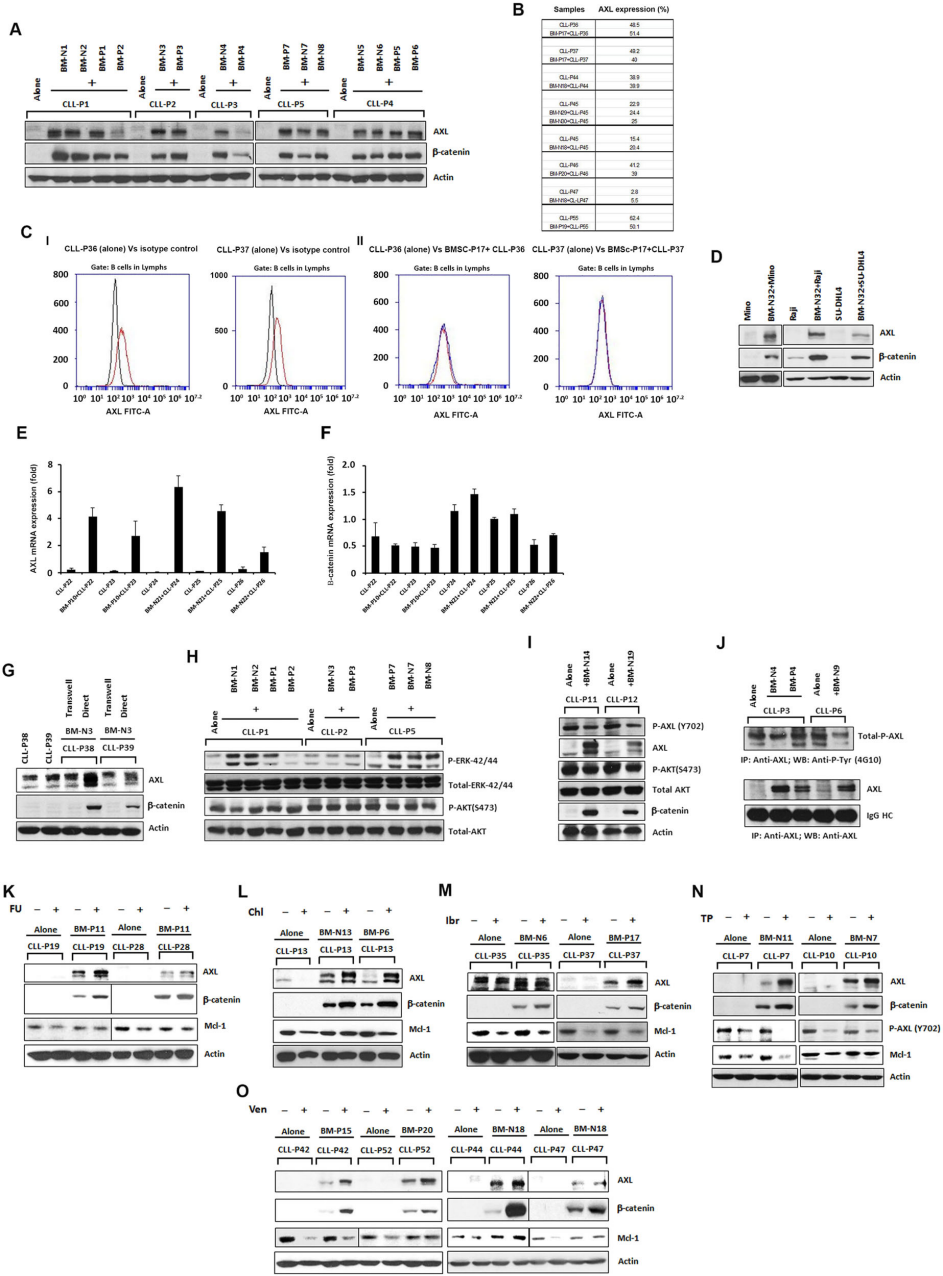
AXL and β-catenin expression and their role in CLL B-cells co-cultured with BMSCs. **A** Increased AXL and β-catenin expression in CLL B-cells. AXL and β-catenin protein levels were determined using separated CLL B-cell lysates after 48 h of co-culture. Actin was used as a loading control**. B** Surface AXL expression on CLL B-cells with or without co-culture with BMSCs. Percent expression of AXL on CD5^+^CD19^+^ B-cells (*n* = 7) surface were determined after 48 h of co-culture with and without with BMSCs (*n* = 6) by flow cytometry. **C** Representative histograms for surface AXL staining on B-cells (red) from two representatives CLL patients (P36, P37) compared to an isotype control (black) (**I**) and when co-cultured with (red) or without (blue) BMSC (P17) (**II**). **D** Co-culture of Mino, Raji, and SU-DHL4 cells with BMSCs. Lymphoma B-cell lysates from Mino or Raji or SU-DHL4 cells co-cultured with or without primary BMSCs were analyzed for the AXL and β-catenin expression by WB analysis. Actin was used as a loading control. **E**, **F** AXL and β-catenin mRNA expressions in CLL B-cells in co-culture with or without BMSCs. AXL and β-catenin mRNA expressions were determined in the CLL B-cells by real time (RT)-PCR. Results are presented as mean values with standard deviation (SD). **G** AXL and β-catenin expressions in CLL B-cells; direct contact versus using transwell. AXL and β-catenin protein levels were examined in CLL B-cell lysates cultured using transwells or in direct contact with BMSCs for 48 h. Actin was used as a loading control **H** Activation of ERK-42/44 in CLL B-cells co-cultured with BMSCs. CLL B-cell lysates were analyzed for the status of P-ERK-42/44 and P-AKT(S473). Total ERK-42/44 and AKT were used as loading controls. **I** P-AXL (Y702) levels in CLL B-cells co-cultured with or without BMSCs. CLL B-cell lysates were examined for the levels of P-AXL(Y702), AXL, P-AKT(S473), AKT, and β-catenin. Actin was used as a loading control. **J** Total tyrosine phosphorylation on AXL in CLL B-cells co-cultured with or without BMSCs. AXL was immunoprecipitated from the CLL B-cell lysates, followed by Western blot analysis using anti-phosphotyrosine (4G10) antibody. The blot was stripped and reprobed with an antibody to AXL. IgG HC was used as a loading control. **K–O** Impact of fludarabine (FU) [3.5 μM], chlorambucil (Chl) [15 μM], ibrutinib (Ibr) [0.75 μM], TP-0903 (TP) [0.15 μM], and venetoclax (Ven) [2.5 nM] on CLL B-cells in co-culture with or without BMSCs. Drugs treated CLL B-cell lysates were analyzed for the levels of P-AXL (Y702), AXL, β-catenin, and Mcl-1. Actin was used as a loading control. CLL patients (P1–P7, P10–P13, P19, P22–P26, P28, P35–P39, P42, P44, P45, P47, P52, P55), normal BMSCs (N1–N9, N11, N13, N14, N18, N19, N21, N22, N29, N30, N32), and CLL BMSCs (P1–P7, P10, P11, P15, P17, P19, P20) are indicated by arbitrary numbers. ‘N’ represents a normal (healthy) control and ‘P’ represents a given patient.

To explore if CLL B-cell/BMSC interaction induces activation of AKT and ERK-1/2 MAPK, post co-cultured leukemic B-cells were analyzed for P-AKT and P-ERK-42/44 by WB. We detected significant increases in P-ERK-42/44 but not in P-AKT(S473) levels in co-cultured CLL B-cells compared to CLL B-cells cultured alone (Fig. 1H). Our further analysis to define AXL activation status, revealed no change in P-AXL (Y702), one of the critical activation sites within the kinase domain of AXL in co-cultured CLL B-cells as compared to CLL B-cells cultured alone (Fig. 1I) despite significant overexpression of total AXL (Fig. 1I). Further total P-AXL levels were determined by immunoprecipitation experiments. Consistent with our findings in Fig. 1I, we also could not detect any significant alteration in total P-AXL level in pre- or post-co-cultured CLL B-cells (Fig. 1J). Therefore, the function of increased AXL levels in co-cultured CLL-B-cells is likely independent of AXL tyrosine kinase activity^5^ and is a subject of our future studies.

Upregulation of AXL and β-catenin is associated with the induction of resistance to multiple chemotherapeutic agents in human cancer cells^6–9^. To see if drug exposure caused further increases in AXL and β-catenin, we treated CLL B-cells with chemotherapy drugs (fludarabine, chlorambucil) used for CLL, at sub-lethal doses (determined from the dose-response curve; Supplementary Fig. 1A) in the absence or presence of BMSCs. After 48 h of fludarabine treatment, significant upregulation of AXL and β-catenin levels were discernible in CLL B-cells in presence of BMSCs as compared to untreated CLL B-cells with or without co-culture with BMSCs (Fig. 1K). In addition, the anti-apoptotic Mcl-1 protein expression level was also partially induced in the presence of BMSCs. Similar observations were also noted when CLL B-cells were treated with a sub-lethal dose (Supplementary Fig. 1B) of chlorambucil for 48 h compared to CLL B-cells without drug treatment in presence of BMSCs (Fig. 1L). Furthermore, treatment of co-cultured CLL B-cells with a sub-lethal dose (Supplementary Fig. 1C, D) of novel agent drugs that included ibrutinib^3^, AXL inhibitor (TP-0903)^3^, or venetoclax also showed upregulation of AXL and β-catenin, over that seen with BMSCs alone (Fig. 1M–O). Thus in vitro drug exposure facilitates further increases in both AXL and β-catenin for co-cultured CLL B-cells consistent with cellular drug resistance.

Since it is known activated ERK and stabilized β-catenin translocate to the nucleus resulting in transcriptional activation of their target genes^10,11^, we subjected CLL B-cells to cytoplasmic/nuclear fractionation following co-culture^12^. Indeed we found increased levels of both active (non-phosphorylated) β-catenin (Ser33/37/Thr41) and P-ERK-42/44 in the nuclear fractions of CLL B-cells co-cultured with BMSCs compared to CLL B-cells cultured alone (Fig. 2A), however, increased AXL expression was only in cytosolic fractions (Fig. 2B). Activated ERK can inactivate GSK-3β via phosphorylation resulting in the accumulation of β-catenin^13^. Here we found significant increases of P-GSK-3β(Ser9) and increases in P-ERK-42/44 and β-catenin expression (Fig. 2C) in co-cultured CLL B-cells. CLL B-cells were also treated with the ERK upstream MEK inhibitor PD98059, in the presence or absence of BMSCs. After 48 h, we found decreases in P-ERK-42/44 level and accompanying decreases in both AXL and β-catenin for CLL B-cells in the presence BMSCs (Fig. 2D). One study reported a positive correlation between c-Jun and AXL expression levels in head and neck squamous cell carcinoma patients^14^. We indeed found increases of P-c-Jun(S73) protein albeit at variable levels in CLL B-cells co-cultured with BMSCs (Fig. 2E). We also detected increased levels of P-c-jun(S73) in Mino, Raji, and SU-DHL4 cells co-cultured with BMSCs versus these cells cultured alone (Fig. 2F). Additionally, treatment of CLL B-cells in co-culture with the c-Jun upstream, JNK inhibitor SP600125, reduced AXL level, and variably β-catenin expression (Fig. 2G). Moreover, there is evidence that AXL can modify β-catenin levels^15^, so we analyzed whether AXL is upstream of β-catenin in Mino cells. We co-cultured Mino cells with BMSCs after being transduced with a lentivirus expressing Cas9 and guide RNAs targeting AXL [as efficient transfection and CRISPR experiment were not feasible in primary CLL B-cells]. CRISPR-mediated reduction in AXL expression in Mino cells did reduce β-catenin expression (Fig. 2H). Overall, these data suggest upregulation of AXL and β-catenin in CLL B-cells is related to the combined effects of c-Jun and ERK activation and that increases in AXL in CLL B-cells are able to further modify β-catenin levels.

**Fig. 2:**
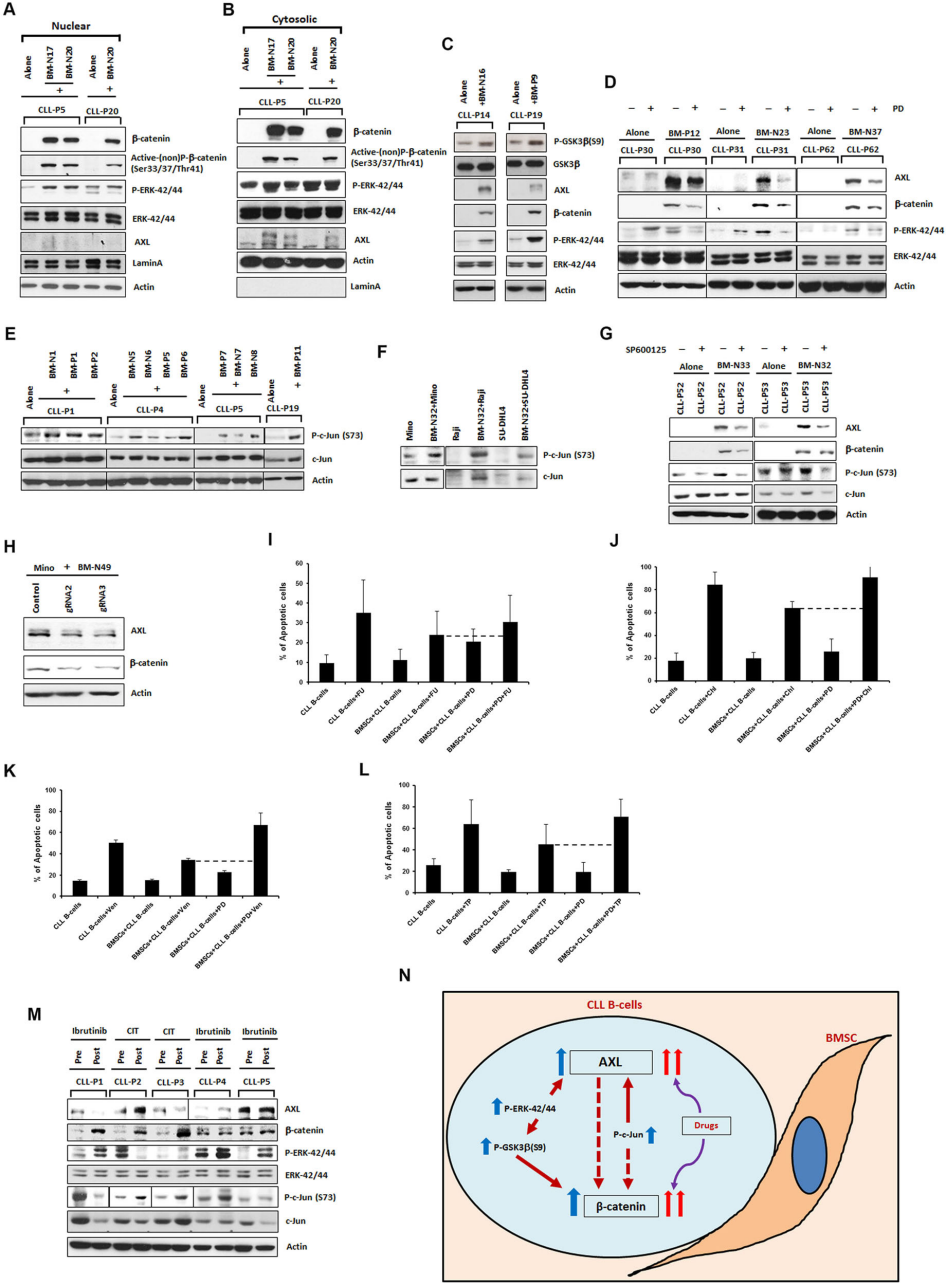
Regulation of AXL and β-catenin expression and role in drug resistance of CLL B-cells in presence of BMSCs. **A**, **B** Expression of β-catenin and P-ERK-42/44 in nuclear and cytosolic fractions of CLL B-cells co-cultured with or without BMSCs. Nuclear and cytosolic fractions from CLL B-cells were analyzed to detect β-catenin, P-β-catenin (Ser33/37/Thr41), P-ERK-42/44, ERK-42/44, and AXL. LaminA and actin were used as loading controls. **C** Increased P-GSK3β in CLL B-cells co-cultured with BMSCs. CLL B-cell lysates were analyzed for the P-GSK3β (Ser9), GSK3β, AXL, β-catenin, P-ERK-42/44, and ERK-42/44. Actin was used as a loading control. **D** Inhibition of ERK-42/44 signaling reduces expression of both AXL and β-catenin in CLL B-cells. PD98059 (70 μM) treated co-cultured CLL B-cell lysates were analyzed for the levels of AXL, β-catenin, P-ERK-42/44, and ERK-42/44. Actin was used as a loading control. **E** Increased c-Jun activity in CLL B-cells co-cultured with BMSCs. CLL B-cell lysates were analyzed for the P-c-Jun (S73) and total c-Jun expression levels 48 h after co-culture. Actin was used as a loading control. **F** P-c-Jun expression in lymphoma B-cells. Lymphoma B-cell lysates from Mino or Raji or SU-DHL4 cells co-cultured with or without primary BMSCs were analyzed for the P-c-Jun (S73) and total c-Jun expression levels. **G** Inhibition of c-Jun activity reduces expression of both AXL and β-catenin in CLL B-cells. SP600125 (15μM) treated CLL B-cell lysates from co-culture were analyzed for the levels of AXL, β-catenin, P-c-Jun(S73), c-Jun. Actin was used as a loading control. **H** AXL regulates β-catenin expression in Mino cells. Mino cells were co-cultured with BMSCs after transduced with control (empty) lentivirus or with virus expressing guide RNAs targeting AXL. Actin was used as a loading control. **I–L** Inhibition of ERK-42/44 activity increases sensitivity towards drug-induced killing of CLL B-cells in co-culture. CLL B-cells treated with the following drugs; fludarabine [FU] (3.5 μM) (**I**) or chlorambucil [Chl] (15 μM) (**J**) or venetoclax [Ven] (2.5 nM) (**K**) or TP-0903 [TP] (0.15 μM) (**L**) alone or in combination with PD98059 [PD] (70 μM) for 48 h in co-culture with BMSCs and induction of cell death was assessed using flow cytometry after staining with annexin/ propidium iodide. Each drug treatment was done with CLL B-cells from two patients in triplicate. Results are presented as mean values with SD. CLL patients (P1, P4, P5, P14, P19, P20, P30, P31, P52, P53, P62), normal BMSC (N1, N5–N8, N16, N17, N20, N23, N32, N33, N37, N49), and CLL BMSCs (P1, P2, P5–P7, P9, P11, P12) are indicated by arbitrary numbers. **M** AXL, β-catenin, P-ERK-42/44, and P-c-Jun expression levels in B-cells from CLL patients. Expression levels were determined on purified leukemic B-cell lysates obtained from CLL patients (*n* = 5) before treatment and while being treated or after treatment. Actin was used as a loading control. ‘CIT’ indicates chemo-immunotherapy. CLL patients (P1–P5) are indicated by arbitrary numbers. ‘N’ represents a normal (healthy) control and ‘P’ represents a given patient. **N** Simplified model of CLL B-cell and BMSC interaction. BMSC induces expression of both AXL and β-catenin in CLL B-cells when co-cultured with BMSCs (blue arrow). Further increase in AXL and β-catenin expression is observed when drugs are added to the CLL B-cell and BMSC co-culture (double red arrows). Elevated level of P-ERK-42/44 in CLL B-cells in the presence of BMSCs, enhances AXL expression and β-catenin accumulation through GSK3β inactivation. Activation of c-Jun in presence of BMSCs also increases AXL expression level and may modulate the increase in β-catenin level (red broken arrow) in CLL B-cells. AXL also may regulate the β-catenin expression (red broken arrow) in CLL B-cells.

To further explore the role of AXL and β-catenin in leukemic B-cell drug resistance, we cultured CLL B-cells alone or with BMSCs in the presence of fludarabine, chlorambucil, venetoclax, and TP-0903 drugs alone or in combination with ERK-42/44 inhibitor PD98059 and measured CLL apoptosis. Co-culture with BMSCs as expected, protected CLL B-cells from drug-induced apoptosis (Fig. 2I–L). However, in the presence of PD98059, which downregulated both AXL and β-catenin expression in CLL B-cells in co-culture (Supplementary Fig. 1E–H), the stromal cell-mediated protection of CLL B-cells was not as effective in suppressing drug-induced killing (Fig. 2I–L). Importantly, PD98059 treatment alone had minimal or no effect on co-cultured CLL B-cell apoptosis (Fig. 2I–L). Moreover, when CLL B-cells not exposed to BMSCs and thus expressing basal levels of both AXL and β-catenin, were treated with fludarabine, chlorambucil, venetoclax, or TP-0903, in the presence of PD98059, there was no enhancement of CLL B-cell sensitivity towards these drugs (Supplementary Fig. 1I). Thus microenvironment mediated signaling via BMSCs leading to increased AXL and β-catenin, enhances drug resistance of leukemic cells.

Finally, we studied CLL B-cells from five CLL patients where we had access to blood samples prior to therapy and then while being treated or after treatment (Supplementary Table 3). We found increases in AXL, β-catenin, P-ERK-42/44, and P-c-Jun(S73) albeit at variable levels, after therapy (Fig. 2M). This finding indicates that AXL and β-catenin presence in CLL B-cells may be a biomarker of drug resistance but further association studies are needed.

In total, our study has found that the interactions between the CLL B-cell and stromal cells in the microenvironment result in modifications of pathways in the leukemic cell known to be associated with drug resistance in human malignancies. Our model highlighting CLL–BMSC interaction and subsequent modification of AXL and β-catenin levels is shown in Fig. 2N. In this current work, we also found that co-culture of human lymphoma cell lines results in enhanced AXL and β-catenin expression suggesting that these biologic phenomena are not limited to CLL B-cells and extend the clinical importance of our findings. Further study of the biology resulting from this cell–cell interaction and relationship to CLL drug resistance for patients on novel agents will add to our knowledge on mechanisms of persistence of disease even in the era of ever more effective therapeutic approaches.

## Financial support

Supported in part by Tolero Pharmaceuticals Inc., Mayo Clinic intramural funding, AKG grant CA170006, and supported in part by the Henry J. Predolin Foundation.

## Supplementary information

SUPPLEMENTAL Materials and Methods

SUPPLEMENTAL Figure legends

SUPPLEMENTAL Table 1

SUPPLEMENTAL Table 2

SUPPLEMENTAL Table 3

SUPPLEMENTAL Figure 1
