# Supplementary material for: Upregulation of AXL and β-catenin in chronic lymphocytic leukemia cells cultured with bone marrow stroma cells is associated with enhanced drug resistance
Source: Blood Cancer J. 2021 Feb 18;11(2):37. doi: 10.1038/s41408-021-00426-2 (PMC7893033; doi:10.1038/s41408-021-00426-2)
Supplement: Supplementary file 1 — SUPPLEMENTAL Materials and Methods [file 41408_2021_426_MOESM1_ESM.docx]

**Supplementary Materials and Methods**

**CLL patients, purification of the leukemic B-cells and cell culture**

All patients studied for blood samples and/or bone biopsy samples provided written informed consent according to the Declaration of Helsinki to the Mayo Clinic Institutional Review Board, which approved these studies. Informed consents were also obtained from healthy donors to obtain their bone biopsy samples. Primary CLL B-cells used for co-culture were purified from blood samples of previously untreated CLL patients (see ***supplementary table 1*** for patient details) using the RosetteSep B-cell enrichment kit (Stem Cell Technologies). The typical purification range of CD5^+^/CD19^+^ CLL B-cells was >95–99% as determined by flow cytometric analysis. CLL B-cells were cultured for optimum viability in serum-free AIM-V (Gibco) medium as previously described (ref. 1).

Primary bone marrow stromal cells (BMSCs) [from age matched healthy donors or CLL patients (*see* ***supplementary table 2***)] were isolated from bone biopsy samples and were maintained in alpha MEM medium (Gibco) supplemented with 10% FBS as previously described (ref. 2).

In some experiments, leukemic B-cells were separated from archived peripheral blood mononuclear cells (PBMCs) stored in liquid nitrogen from CLL patients both before, during and after treatment (see ***supplementary table 3*** for patient details) using EasySep Human B-cell enrichment kit II without CD43 depletion (StemCell Technologies) when needed.

**Reagents**

TP-0903, a high-affinity AXL inhibitor, was a kind gift from Tolero Pharmaceuticals. Ibrutinib (PCI-32765) and venetoclax were purchased from Selleck Chemical; whereas fludarabine and chlorambucil were from Sigma. Phosphotyrosine antibody 4G10 was from Millipore, β-catenin and Lamin A were from Santa Cruz Biotechnologies. FITC–conjugated secondary antibody to rabbit IgG was from R&D Systems, fluorescence-conjugated antibodies to CD5, CD19 were purchased from BD Biosciences and SP600125 (JNK inhibitor) was purchased from Tocris Bioscience. MEK inhibitor PD98059 and all other antibodies were purchased from Cell Signaling Technologies.

**Co-culture of CLL B-cells with BMSCs**

For co-culture experiments, primary BMSCs were seeded at 1 × 10^6^ cells/10 mL of alpha-MEM medium and cultured until the cells were approximately 80% confluent. Primary CLL B-cells were isolated and purified from blood of previously untreated patients with CLL. After washing, BMSCs were co-cultured with purified primary CLL B-cells at a cell density of 1:50 ratio in serum-free AIM-V medium. After 48h, CLL B-cells were harvested after gentle agitation by removal of the supernatant. The migrated fraction of CLL B-cells (CD45+) beneath BMSCs (CD45-) which occurs during co-culture was recovered by using a human CD45 depletion kit (EasySep). CLL B-cell fractions (harvested and migrated fractions) were then pooled together for further analysis. CD45- BMSC fractions were also collected for further analysis. CLL B-cells were also co-cultured with BMSCs at a ratio of 50:1 separated by a transwell (0.4 µm-pore size; Becton Dickinson) for 48h.

**Co-culture of Mino, Raji and SU-DHL4 cells with BMSCs**

All lymphoma cell lines (purchased from ATCC), were maintained in RPMI medium (Gibco) supplemented with 10% FBS. For co-culture experiments, primary BMSCs were seeded at 1 × 10^6^ cells/10 mL of alpha-MEM medium and cultured until the cells were approximately 80% confluent. After washing, BMSCs were co-cultured with Mino, Raji or SU-DHL4 cells at a cell density of 1:20 ratio in RPMI medium supplemented with 10% FBS. After 48h, Mino or Raji or SU-DHL4 cells were harvested after gentle agitation by removal of the supernatant. The migrated fraction of B-cells (CD45+) beneath BMSCs (CD45-) was recovered by using human CD45 depletion kit (EasySep) as described above. B-cell fractions were then pooled together for further analysis. CD45- BMSC fractions were also collected for further analysis.

**CLL B-cells cultured with BMSCs in the presence and absence of drugs**

BMSCs were seeded at 1 × 10^6^ cells/ 10 mL and cultured until the cells were ~80% confluent. After washing, BMSCs were co-cultured with CLL B-cells at a 1:50 ratio in serum-free AIM-V medium. The co-cultured CLL B-cells were then treated with DMSO or the following sub lethal drug concentrations; 3.5μM of fludarabine, 15μM of chlorambucil, 0.75μM of ibrutinib, 15μM of SP600125 and 70μM of PD98059 as needed for 48h. TP-0903 (0.15μM) and venetoclax (2.5 nM) treatments were done for 24h after 24h of initial co-culture. For comparison, CLL B-cells were cultured alone and treated similarly with DMSO or the above mentioned drugs. After 48h, CLL B-cells and BMSCs were separated and harvested and cell lysates were prepared in RIPA lysis buffer (Boston Bio Products) for further analysis.

**Nuclear and cytosolic protein extraction of CLL B-cells**

CLL B-cells recovered after 48h of co-culture with BMSCs, were incubated with hypotonic buffer [10mM HEPES (pH 7.8), 10mM KCl, 2mM MgCl2, 0.1mM EDTA, 10μg/ml aprotinin, 3mM dithiothreitol (DTT), and 0.2mM phenylmethylsulfonyl fluoride(PMSF)] for 15 min on ice. Nonionic detergent 10% IGE-PAL (Sigma Aldrich) was then added to the cell suspension and mixed vigorously. Thereafter, the whole mixture was centrifuged at 14,000 rpm for 5 min. Supernatant was collected as the cytoplasmic fraction and stored for experiments. Collected pellets were then suspended in a hypertonic buffer solution (50mM HEPES pH 7.8, 50mM KCl, 300mM NaCl, 0.1mM EDTA, 10μg/ml aprotinin, 3mM DTT, and 0.2mmol/l PMSF) and mixed on a rotating rack for 25 min at 4°C. Finally, the sample was centrifuged at 20,000 g for 10 min, and the supernatant was collected as the nuclear extract.

**CRISPR-cas9 mediated knockdown and co-culture experiment**

Three different single guide RNA (sgRNA) targeting AXL containing lentiCRISPR v2 plasmid was obtained from Genscript. Lentiviral plasmids were co-transfected into HEK293T cells with packaging vectors psPAX2 (Addgene plasmid; a gift from Didier Trono) and pMD2.G (Addgene plasmid; a gift from Didier Trono) using PEI MAX™ transfection Reagent (Polysciences). Virus-containing medium was collected 48h after transfection and cleared of potential cells using 0.45-μm Steriflip filter units (Millipore Sigma). Lentivirus was mixed with polybrene (final concentration 8 µg/mL; Millipore Sigma) to transduce Mino cells. Twenty-four hours after infection, cells were treated with 0.25 µg/mL puromycin-containing medium. Forty-eight hours after transduction, Mino cells were used for co-culture with BMSCs. After 48h of co-culture, Mino cell lysates were prepared followed by Western blot analysis to detect relevant proteins using specific antibodies.

**Immunoprecipitation and Western blot analysis**

For the immunoprecipitation (IP) experiments, 0.2–0.3mg of CLL B-cell lysates were incubated with 2μg of specific antibody for 4h, followed by addition of 30μl of CHIP grade agarose beads (Cell Signaling), overnight at 4°C. Beads were washed, digested in Laemmli buffer and subjected to Western blot analysis using specific antibodies.

**Real- time-polymerase chain reaction (RT-PCR)**

Total cellular RNA was extracted from purified CLL B-cells from co-culture with BMSCs using PureLink RNA Mini Kit (Ambion) and 0.5μg of total RNA from each sample was reverse-transcribed using the SuperScript® III First Strand Synthesis Kit (Invitrogen) according to the instructions of the manufacturer. RT-PCR was performed using the SYBR Green PCR core Master Mix (Applied Biosystems, Foster City, CA) on a 7500 Real-Time PCR System (Applied Biosystems) according to the manufacturer’s instructions. Comparative real-time PCR was performed in triplicate. Relative expression was calculated using the comparative Ct method (ref. 3) and presented here as “fold expression. GAPDH was used for cDNA normalization. AXL, β-catenin and GAPDH primers were purchased from SABiosciences.

**Determination of AXL expression on CLL B-cell surface by flow cytometry**

Purified primary CLL B-cells (1 x 10^6^) with or without co-culture with BMSCs for 48h were washed and incubated with fluorochrome conjugated mouse monoclonal antibodies to CD5 and CD19 in presence or absence of 1.5 μl of isotype control or anti-AXL rabbit antibody (unconjugated; Cell Signaling) for 30 min in dark at room temperature. Cells were washed in PBS, then we added anti-rabbit IgG-FITC to the cells and incubated at 4°C in dark for 30 min. Cells were washed, fixed in 1% paraformaldehyde solution and then analyzed by flow cytometry (BD Accuri C6 Plus).

**Cytotoxicity assay to determine the drug sensitivity of CLL B-cells**

CLL B-cells (2X10^6^cells/mL) from previously untreated patients were treated with increasing doses of fludarabine/chlorambucil/ibrutinib/venetoclax in serum-free AIM-V medium. Cells were harvested, and induction of apoptosis was determined by flow cytometry (BD Accuri C6 Plus) after staining with annexin/ propidium iodide (PI) and then dose response curves were generated. Next CLL B-cells cultured alone or co-cultured with BMSCs at a 50:1 ratio in serum-free AIM-V medium. CLL B-cells were then treated with 70μM of the ERK inhibitor PD98059 and then added DMSO as a control or the following sub lethal drug concentrations; 3.5μM of fludarabine and 15μM of chlorambucil for 48h. TP-0903 (0.15μM) and venetoclax (2.5 nM) treatments were done for 24h after 24h of initial culture in presence or absence of BMSCs with or without PD98059 treatment. After 48h, cells were harvested, and induction of apoptosis (early apoptotic, late apoptotic and dead cells) was determined by flow cytometry (BD Accuri C6 Plus) after staining with annexin/ PI and presented as a sum of all three fractions as “% Apoptotic cells”.

**References**

1. Sinha S, et al. Targeted Axl Inhibition Primes Chronic Lymphocytic Leukemia B Cells to Apoptosis and Shows Synergistic/Additive Effects in Combination with BTK Inhibitors. Clin Cancer Res. 2015;21(9):2115-26.

2. Kay NE, et al. Bone biopsy derived marrow stromal elements rescue chronic lymphocytic leukemia B-cells from spontaneous and drug induced cell death and facilitates an "angiogenic switch". Leukemia Research. 2007;31(7):899-906.

3. Pichiorri F, et al. MicroRNAs regulate critical genes associated with multiple myeloma pathogenesis. Proc Natl Acad Sci U S A. 2008;105(35):12885-90.
