# Supplementary material for: Upregulation of AXL and β-catenin in chronic lymphocytic leukemia cells cultured with bone marrow stroma cells is associated with enhanced drug resistance
Source: Blood Cancer J. 2021 Feb 18;11(2):37. doi: 10.1038/s41408-021-00426-2 (PMC7893033; doi:10.1038/s41408-021-00426-2)
Supplement: Supplementary file 2 — SUPPLEMENTAL Figure legends [file 41408_2021_426_MOESM2_ESM.docx]

**Supplementary figure legends**

**Figure 1: A, B, C and D. Dose response curves.** CLL B-cells from previously untreated patients (n=19) were treated with increasing doses of fludarabine for 72 hours (**A**). CLL B-cells from previously untreated patients (n=12) were treated with increasing doses of chlorambucil for 48 hours (**B**). CLL B-cells from previously untreated patients (n=3) were treated with increasing doses of ibrutinib for 48 hours (**C**). CLL B-cells from previously untreated patients (n=13) were treated with increasing doses of venetoclax for 24 hours (**D**). CLL B-cell death was determined by flow cytometry after staining with annexin/propidium iodide. Results are presented as mean values with SD at each dose of drugs. The mean LD_50_ value is indicated by the red dashes. **E, F, G and H.** **Inhibition of ERK-42/44 activation in CLL B-cells co-cultured with BMSCs inhibits AXL and β-catenin expression.** CLL B-cells were treated with fludarabine [FU] (3.5μM) or chlorambucil [Chl] (15μM) or venetoclax [Ven] (2.5nM) or TP-0903 [TP] (0.15μM) alone or in combination with PD98059 [PD] (70μM) in co-culture with BMSCs were analyzed by WB analysis for the AXL, β-catenin, P-ERK-42/44 and ERK-42/44 expression levels. Actin was used as a loading control. One representative blot is shown for each drug treatment. CLL patients (P63, P64, P66, P67), normal BMSC (N45) and CLL BMSCs (P15, P24, P26) are indicated by arbitrary numbers. ‘N’ represents a normal (healthy) control and ‘P’ represents a given patient **I. Impact of ERK-42/44 signaling on drug-induced killing of CLL B-cells.** Previously untreated CLL B-cells alone treated with fludarabine [FU] (3.5μM) or chlorambucil [Chl] (15μM) or TP-0903 [TP] (0.15μM) or venetoclax [Ven] (2.5nM) alone or in combination with PD98059 [PD] (70μM) for 24h or 48h and were then analyzed by flow cytometry after staining with annexin/propidium iodide to determine the level of CLL B-cell death. One representative plot is shown of three independent experiments. CLL patient (P68) is indicated by arbitrary number. ‘P’ represents a given patient.
