# Supplementary material for: Upregulation of AXL and β-catenin in chronic lymphocytic leukemia cells cultured with bone marrow stroma cells is associated with enhanced drug resistance
Source: Blood Cancer J. 2021 Feb 18;11(2):37. doi: 10.1038/s41408-021-00426-2 (PMC7893033; doi:10.1038/s41408-021-00426-2)
Supplement: Supplementary file 4 — SUPPLEMENTAL Table 2 [file 41408_2021_426_MOESM4_ESM.docx]

**Supplementary Table 2: Characteristics of untreated CLL patient cohort used to isolate BMSCs for co-culture study**

**P**; Untreated CLL patients, **ND**; Not done
