# Supplementary material for: Upregulation of AXL and β-catenin in chronic lymphocytic leukemia cells cultured with bone marrow stroma cells is associated with enhanced drug resistance
Source: Blood Cancer J. 2021 Feb 18;11(2):37. doi: 10.1038/s41408-021-00426-2 (PMC7893033; doi:10.1038/s41408-021-00426-2)
Supplement: Supplementary file 5 — SUPPLEMENTAL Table 3 [file 41408_2021_426_MOESM5_ESM.docx]

**Supplementary Table 3: Characteristics of CLL patient cohort studied before, during and after treatment**

**ND**; Not done, **Ofa**: Ofatumumab, **Pento**: Pentostatin, **Cy**: Cyclophosphamide, **Benda**: Bendamustine, **Ritu**; Rituximab
