# Supplementary figures and images for: Upregulation of AXL and β-catenin in chronic lymphocytic leukemia cells cultured with bone marrow stroma cells is associated with enhanced drug resistance
Source: Blood Cancer J. 2021 Feb 18;11(2):37. doi: 10.1038/s41408-021-00426-2 (PMC7893033; doi:10.1038/s41408-021-00426-2)

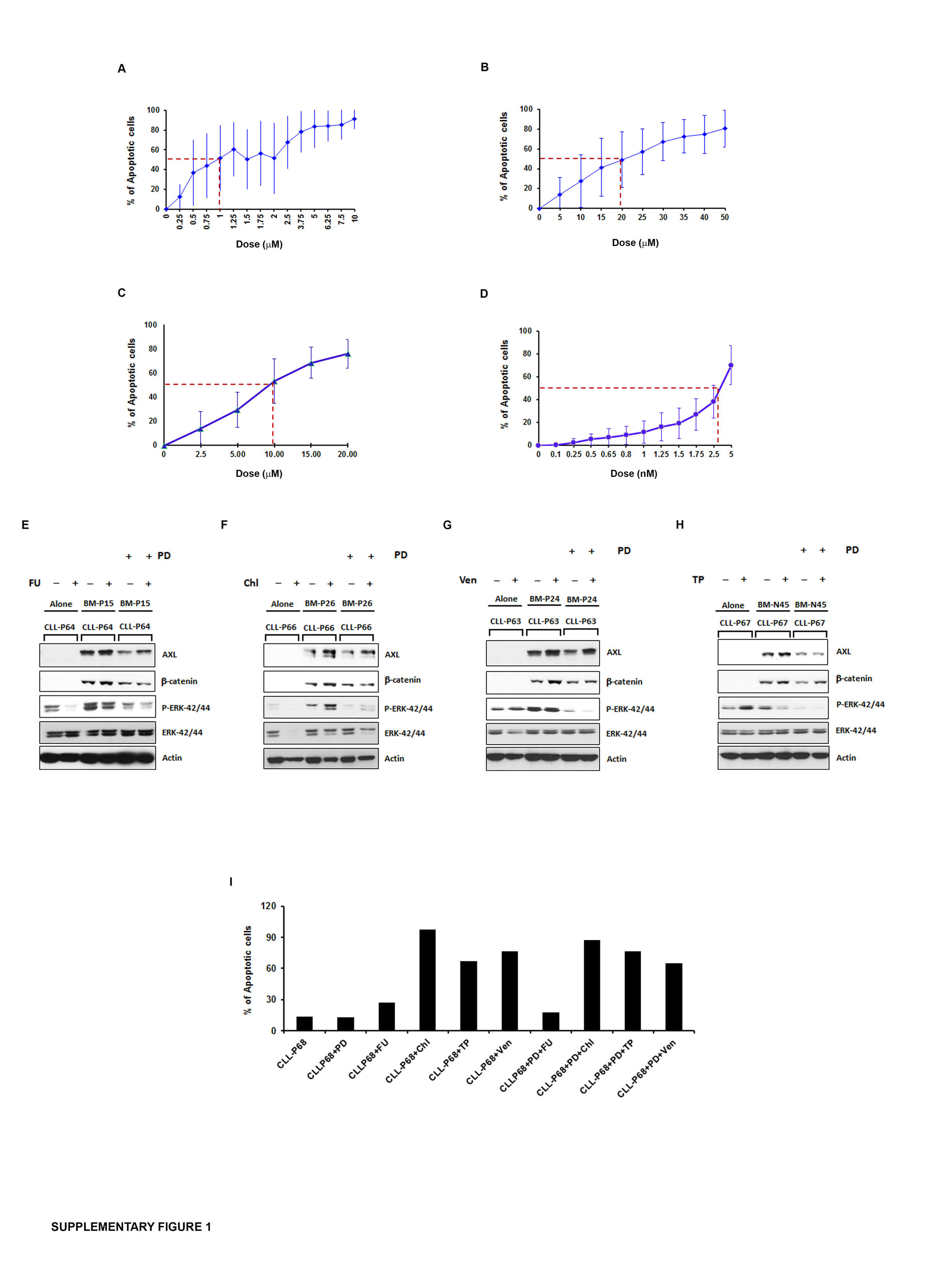

Supplement: Supplementary file 6 — SUPPLEMENTAL Figure 1 [file 41408_2021_426_MOESM6_ESM.jpg]
